# Supplementary material for: An improved reduced-order model for pressure drop across arterial stenoses
Source: PLoS One. 2021 Oct 1;16(10):e0258047. doi: 10.1371/journal.pone.0258047 (PMC8486142; doi:10.1371/journal.pone.0258047)
Supplement: S1 Appendix — Explanation of the second term derivation in the reduced order model. (PDF) [file pone.0258047.s001.pdf]

1 S1 Appendix: Derivation of turbulence term

For simplification, we define alternative notations here. The Navier-Stokes velocity field and strain rate tensor is denoted with u_i, s_{ij} and the corresponding quantities in Stokes flow with capital letters, U_i, S_{ij}.

We have the term $u_i u_j S_{ij}$ to model which is based on the Navier-Stokes field u_i, u_j and the Stokes field S_{ij}. Since the instantaneous velocities and strain rate tensor is impossible to simplify, we are interested in modelling these term when the flow is time-averaged. In a similar way Reynolds-Averaged-Navier-Stokes modelling is derived, we regard that each instantaneous quantity is composed of an average value $\bar{()}$ and a fluctuating value $^{(')}$. The term is thus decomposed as

$$u_i u_j S_{ij} = \overline{u_i u_j S_{ij}} + (u_i u_j S_{ij})^{'} \tag{1}$$

The time-averaged term is

$$\begin{aligned} \overline{u_i u_j S_{ij}} &= \overline{u_i u_j}\,\overline{S_{ij}} + \overline{(u_i u_j)' S_{ij}^{'}} = \\ &= \overline{(\bar{u}_i + u_i^{'})(\bar{u}_j + u_j^{'})}\,\overline{S_{ij}} + \overline{(u_i u_j)' S_{ij}^{'}} = \\ &= (\bar{u}_i \bar{u}_j + \overline{u_i^{'} u_j^{'}})\overline{S_{ij}} + \overline{(u_i u_j)' S_{ij}^{'}} = \\ &= (\bar{u}_i \bar{u}_j)\overline{S_{ij}} + \overline{u_i^{'} u_j^{'}}\,\overline{S_{ij}} + \overline{(u_i u_j)' S_{ij}^{'}} \end{aligned} \tag{2}$$

Putting together the above two equations, the quantity in the integral of the second term in the pressure drop equation (the "turbulent" term) becomes:

$$u_i u_j S_{ij} = (\bar{u}_i \bar{u}_j)\overline{S_{ij}} + \overline{u_i^{'} u_j^{'}}\,\overline{S_{ij}} + \overline{(u_i u_j)' S_{ij}^{'}} + (u_i u_j S_{ij})^{'} \tag{3}$$

1. The first term on the RHS of Eq.3 is zero since the product of the velocities in either directions x,y,z is of $\mathcal{O}(10^{-4})$ for terms involving the radial velocity components and $\mathcal{O}(10^{-2})$ or less for the velocity at the flow direction. The most significant contribution of the term is at the flow direction according to Ji et al, 2015, which might be of $\mathcal{O}(10^{-4})$ for coronary artery flows.
2. The third term on the RHS of Eq.3 is negligible, if we assume that the fluctuation of $S_{ij}^{'}$ in Stokes flow is non-significant.
3. The fourth term on the RHS of Eq.3 is negligible, if we consider that the fluctuating part of the product of the velocities $(u_i u_j S_{ij})^{'}$ is relatively small.
4. The only thing remaining is to consider that the strain rate tensor of the Stokes flow is approximately equal to the strain rate tensor of the Navier-Stokes, e.g. $S_{ij} \approx s_{ij}$. This is done for including all terms in the integral from the Navier-Stokes system of equations and further simplifying the term with a quantity with a physical meaning: turbulence dissipation. This assumption is expected to be valid for lower Re, but for higher Re it might not be valid.

Considering all the above,

$$u_i u_j S_{ij} \approx \overline{u_i^{'} u_j^{'}}\,\bar{s}_{ij} \tag{4}$$

This is equal to the turbulent kinetic energy production which is equal to the opposite of ϵ, the turbulent kinetic energy dissipation.
