# Supplementary material for: An improved reduced-order model for pressure drop across arterial stenoses
Source: PLoS One. 2021 Oct 1;16(10):e0258047. doi: 10.1371/journal.pone.0258047 (PMC8486142; doi:10.1371/journal.pone.0258047)
Supplement: Attachment — Submitted filename: Response_to_Reviewers.docx [file pone.0258047.s002.docx]

Please use the space provided to explain your answers to the questions above. You may also include additional comments for the author, including concerns about dual publication, research ethics, or publication ethics. (Please upload your review as an attachment if it exceeds 20,000 characters)

Reviewer #1: This manuscript presented a new formulation based on a consistent theoretical analysis of the NS equations. Numerical simulations are used to validate the proposed formula, showing that the new formula produces more accurate results than other existing models. This manuscript is well written and organized, I have some concerns as follows:

1. It seems that the manuscript is focusing on the development and validation of a new model, which is basically a formula, for the prediction of pressure drop. I feel it does not fit the current title "numerical investigation of ...". The numerical simulation is only a way to validate the proposed model.

*We appreciate the reviewer’s comment and have revised the title to “An improved reduced-order model for pressure drop across arterial stenoses”*

2. The authors claimed that the proposed model can handle turbulence better with the new term, I would like to see more discussion in terms of this point.

*In relation to the turbulence term, we summarise here the discussion points made in our original submission:*

- *The physical origin of the quadratic term is ambiguous and is likely an empirical choice (L53)*
- *There is evidence that turbulence effects contribute to pressure loss in high and low Re flow in arteries (L59)*
- *We have assumed the physically unspecific term is mostly due to turbulence, and modified the formuation explicitly based on modelling turbulent energy dissipation (L211 onwards)*
- *Contrary to other ROMs which tend to overestimate the contribution of the turbulence term, our model does not (L354-359, also Fig 5-i). Further observation of the individual terms in models of Young and Itu together show that the overestimation is not attributable to the other terms (L370 onwards).*

*Based on these points, and considering the improved results of the proposed model, we believe our evidence supports the claim. We regret that this was not plainly stated and have added further discussion at L501 in a new section entitled Model Evaluation.*

3. The comparisons presented here are all assuming the presented numerical results as the accurate one, which is not convincing, I believe comparisons considering benchmark cases or data from others are necessary.

*The accuracy of the numerical simulations is a subject we have given careful consideration to. Prior to conducting this study, we performed a systematic comparison of four CFD solvers applied to a range of benchmark arterial stenoses problems. The solvers evaluated include OpenFOAM (Weller et al., 1998), Nektar++ (Cantwell et al, 2015), CHeart (Lee et al., 2016) and Crimson (Arthurs et al, 2021). Collectively, they employ finite element, finite volume, and spectral h/p methods, with monolithic or operator-splitting approaches and fully implicit or hybrid time stepping algorithms. Convergence analysis revealed that despite the varying numerical approaches, the agreement in* $\Delta p$ *among the solvers and with experiments was very good as demonstrated in the table below (<0.1% in axisymmetric lesion):*

*Importantly, the level of discrepancy among the numerical solutions in the classes of geometries used in the current work is order(s) smaller compared to the typical difference between the ROMs (shown in Fig 2 to 5 of the manuscript). Based on these findings, we believe our choice of the numerical solver was not a significant source of error for the results presented.*

*In evaluating the model in comparison to other studies, we employ additional data from the study of Ventre et al. (2019) and the in-vivo artery measurement from Young et al. (1975), in addition to the in vitro experimental data already used in the original submission. Ventre et al. compared 0D (Young’s model), 1D and 2D (multi-ring) formulations with the experimental measurement and found that the 0D and 2D formulations were most accurate in the low and high severities, respectively. Their original plot has been adopted below. Adding our modified 0D model and 3D CFD simulations to this benchmark problem shows that both of these approaches estimate the measurements more closely across the range of stenosis severities, and that the current ROM formulation is also more accurate than the previously improved model of Itu et al. (2016).*

*These points have been added to the new discussion section* *Model Evaluation (L501).*

4. The Re is not defined in the manuscript.

*We thank the reviewer for pointing out this omission and we have added its definition in page 2.*

5. The proposed model uses flow rate instead of velocity, why and what is the difference.

*Volumetric flow rate is straightforwardly converted to velocity using the cross-section area; in the design of ROMs, it is widely used not only in our model but in almost all historical formulations proposed. The flow rate Q tends to arise naturally when modelling the different terms of the formula and simplifies the way in which mathematics are expressed. We could have just as easily divided both sides of our formula by the area to get it in terms of velocity, but this would result in an unnecessary complexity.*

Reviewer #2: The work presents a new reduced-order model for pressure drop across the stenosis. This model technically sounds and is attractive.
Unfortunately, the design of this study is inappropriate. The authors apply their findings to the FFR calculations, i.e. the pressure drop evaluation in the stenosed coronary arteries. The authors compare their reduced order model with the 3D CFD flow in a single tube with rigid walls, sinus-like inflow velocity profile and zero gradient outflow boundary conditions. They demonstrate excellent agreement with this model. Unfortunately, the reference CFD model is entirely unrealistic. Essential features of the coronary circulation and FFR evaluation are the constant myocardium contractions, flexible walls, network structure of the vascular network, the change in the terminal resistance due to hyperemia due to the vasodilator administration. All these features are important but beyond the reference model. Thus it is not a surprise that the authors failed to reproduce the findings of the other works on virtual FFR evaluation.

*We thank the reviewer for these comments and regret that we have not been clearer in defining the broader context of this work in our initial submission. The model we are developing in this paper is intended for one component within a multiscale ensemble of models used to characterise a vascular system. As each of the governing models have differing dimensionalities and operate drastically differently, it is natural to try and understand its behaviour under well-defined, controlled conditions with as few confounders as possible. Every multiscale model setup is unique and has their own unique sources of error. Evaluations exclusively based on multiscale settings have left unanswered questions on some of the most well-known ROMs:*

- *Prior to our work, we saw in the literature evaluations of ROMs within multiscale models that disagreed strongly – why? The model of Young et al. (1973) was shown to be highly accurate in Blanco et al. (2018) and Müller et al. (2019), but grossly inaccurate in Itu et al. (2013) and Mirramezani et al. (2019) even though all studies employed multiscale network models based on patient data.*
- *Logic dictates that if a model works well under complex settings, it should also work when we take away the complexity. If not, would it not suggest its constituent terms are incorrectly modelled? Though ROMs are phenomenological, each term still represents a distinct flow mechanism. The model of Itu et al. (2013) has been shown to work well under multiscale settings, but our vessel-level tests show a drop in performance.*

*In order to answer these questions, we believe the complexities need to be peeled back, rather than layers added.*

*A complete evaluation of a ROM should involve testing in a network setting as well as using patient data. However, vessel-level evaluation should not be skipped; often the overhead of constructing multiscale models makes opaque whether the 0D component has been systematically exposed to a sufficiently broad range of testing conditions:*

- *In studies using patient-imaging data, to correctly sample the patient variability the number of patients must be very large. It is onerous to construct so many multiscale models, moreover, patient selection is often driven by availability instead of data imbalance.*
- *Due to space limitation, important parameter distributions are often masked under a wealth of detail in these reports. As a reader often it is difficult to assess the parameters of the individual stenoses simulated, flow/pressure wave forms in those vessels, etc.*
- *Also, where an artificial lesion is inserted for investigation, the site of the lesion tends to remain unchanged, narrowing the range of flow condition the lesion is exposed to.*

*Our tests are designed to sample widely varying contributions of different terms composing ROMs. The problem set up has yielded cases which are viscous-dominated (fig 3), inertia-dominated (fig 2, 4), and balanced with increased turbulence contribution (fig 5). Where we lack in physiological realism at this stage, we make up for by controlling the test samples explicitly across the physiological distribution.*

*From a broader physiological perspective, the features of coronary circulation mentioned are indeed essential for systemic function. However, when one considers specifically what factors determine trans-stenotic pressure drop, there is far more room to strip the physics bare down to the basics:*

- *Elastance - simulations studies employing elastic walls consistently report the irrelevance of elastance in pressure drop. Please refer to our further response in #5.*
- *Network geometry - from what we know of PDEs, the solution field of a subdomain remains unchanged if the original solution is imposed as boundary condition on the sub-boundary. Indeed, it is standard practice to use ROMs to approximate the distal boundary conditions. However, this principle has been demonstrated on more discrete settings as well; In Epstein et al. (2015), vascular network models were systematically truncated up by absorbing the distal segments into the windkessel parameters. It was found that even at around 70% reduction in the number of segments, the pressure waveforms may be affected only by around 4%. As long as we can recreate similar flow conditions in the vicinity of the lesion, what happens elsewhere in the network is of little concern to testing the stenosis model. (Please also refer to our survey of vFFR literature in response #2).*
- *Hyperaemia - in most studies, a fixed ratio of distal resistance reduction (Total Coronary Resistance Index) is used to model the effects of it, resulting in an increased flow rate. Such an approach is important when making predictions of the unknown, but unnecessary when mapping out a local lesion model behaviour and only adds unclarity. In our work we directly apply a high flow rate to simplify the interpretation.*

*Finally, the trend in FFR_CFD_ of using a multiscale model has been popularised for applications in CCTA starting with Kim et al. (2010), and has been successful for making predictions not least due to well-established methods that exist for tuning parameters of the sub-systems from imaging data (e.g. terminal resistances from allometric scaling, LV mass, HR, EF, etc). Since these can be determined on a patient-specific basis, and with commercial offerings well-established in the market (Heartflow), we regard the CT-based FFR problem as having a matured solution.*

- *However, in other applications (key example being x-ray angiography) these patient parameters cannot readily be determined and thus require us to pose assumptions, which become liabilities rather than insights for diagnostic prediction.*
- *Most, if not all, of the angio-vFFR trials that have appeared in the literature in recent years (please refer to our response to #2) model a single coronary segment at a time equipped with a lighter simulation set up. For better or worse, this isn't going to change given the nature of the data, and optimal modelling approaches should be sought within these constraints.*

*Please find our specific revisions pertaining to these points in the below case-by-case response.*

I suggest the following todo list before the future submissions:
1) to study recent reviews on atherosclerosis modelling by a reduced-order approach;

*We have addressed this suggestion and widened our discussion of the relevant literature. Keeping our focus on algebraic ROMs we did not find papers devoted solely to reviewing ROMs within the recent years, however 2 studies contained a comparative analysis of ROMs as part of their investigation (Mirramezani et al. 2019; Ventre et al. 2019), and others which employed alternative approaches to reduced-order modelling, which we briefly review below.*

***Mirramezani et al, 2019****: A multiscale comparative study of 0d/1d/2d/3d models of multivessel coronary tree, based on a representative CCTA dataset and 3 patient-specific datasets. Tested 4 ROMs within this set up (Garcia et al., 2005; Seely & Young, 1976; Itu et al., 2013 and Huo et al, 2012) for axisymmetric and asymmetric stenoses, as well as 1D/2D models against 3D CFD, where 0D models were shown to outperform both 1D and 2D. For this, the flow rates were calculated by 3D CFD and plugged into 0D model. Similar to our own results, the model of Itu performed better than Young’s (Seeley & Young, 1976) and also consistent with our observation, Young’s model tended to frequently overestimate the pressure drop.*

***Ventre et al., 2019****: 2D (Multi-Ring), 1D and 0D (Young’s) models were compared in idealised geometries of aortic coarctation. Additionally, it used a gradient-based optimiser to fit the best empirical coefficients for the 0D model to approximate the unsteady 1D/2D results, thereby testing their optimal modelling capacity. This improved the performance of 0D model, as is expected given the functional similarities between Young and Itu models (which largely only differ by how the coefficients are modelled). Using this comparison as a benchmark, we have shown our proposed model to outperform both Young and Itu (please refer to our response #3 of reviewer 1).*

***Kim et al., 2020****: Proposed a modelling strategy that uses a combination of converging/straight pipe ROMs for the pre-throat section and machine learning in the diverging section, in acknowledgment of the difficulties associated with flow separation/secondary flow/turbulence effects. Validation featured 33 CCTA stenosis geometries from 31 patients but no FFR was measured, and 0D results were compared to 3D CFD. Our proposed model addressed the same shortcomings in the diverging section but is free from the constraints of machine learning (need for carefully compiled training data to avoid bias, inability to extrapolate, blackbox-ness).*

***Schrauwen et al., 2015****: This approach proposed a polynomial function approximation of the velocity profile and fitted its parameters to the CFD results, then used it to predict pressure drop. The final model is essentially a 1D NS, numerically solved as a single 1D integral. 10 straightened IVUS geometries were used for validation, which contained mild stenoses mostly (only 2 lesions above 70% severity) and the prediction accuracy at deteriorated higher Re. This approach did not outperform 0D ROMs.*

***Buoso et al., 2019****: This contrasting approach to ROM does not use a 0D formulation, but an online-offline approach where 3D CFD (offline) solutions are projected on to low-dimensional subspace using Proper Orthogonal Decomposition. During prediction (online) phase, discrete empirical interpolation method (DEIM) is used to derive a ROM approximating the NS operators. All tests were done in a straight idealised pipe model.*

*There were other studies which employed 1D or 2D multi-ring models directly to calculate trans-stenotic pressure drops without an auxiliary lesion model; these approaches, while within the definition of being reduced-order, were not considered due to difference in scope pursued in our paper (Some are discussed in our response #2). As previous studies have reported, 0D algebraic models, when tuned properly is capable of* $\Delta p$ *prediction that is bested only by 3D CFD (Mirramezani et al., 2019; Ventre et al, 2019), and in our opinion provides an optimal balance of accuracy with computational cost over alternative approaches in ROM. These points have been added to the now-expanded Modelling Approaches section in the Discussion (L522).*

2) to study recent works on virtual FFR evaluation by reduced order approaches, especially patient-specific modelling with (at least) acceptable statistical accuracy (A lot of contemporary works demonstrate suitable correlation with medical data.);

*The recent work on computationally-derived FFR split into largely two bodies of literature; by whether they apply FFR modelling on CCTA or on x-ray angiography data. As both are patient-specific yet with very different modelling choices, we have organised our below mini-survey by this division.*

***FFR_CT_***

*Most recent studies in this category employ a 1D model to address the flow in the coronary tree, and many of them additionally use a 0D ROM to characterise the pressure drop across the lesion. Several papers were focused on sophisticated methodology and patient-specific model components. Since no functional (pressure, flow) measurements were available, various schemes for assigning BCs were used. Most have used patient anatomical data (proximal coronary tree), but only some with measured FFR, in offline retrospective analyses. Most studies feature a small patient number that is statistically underpowered.*

***Carson et al., 2019****: A benchmark study of FFR_CT_ workflows. Four groups participated (3 1D and 1 rigid wall 3D CFD models, where all but one 1D model additionally used a 0D stenosis ROM). 10 cases of patient CT/FFR measurements were used and shared online. It was the pure 1D model (without a stenosis ROM) that yielded best agreement with clinical FFR measurements; on the other hand, only 2 of the 14 FFRs were positive (i.e. <0.8), and the rest were mild lesions.*

***Boileau et al. 2017****: This is the earlier work from Swansea group that led the Carson paper. They undertook a 3D vs (1D+0D) comparison, highlighting a nonzero bias in the estimated FFR by the 1D+0D model. Subsequently the friction term and viscoelastic wall law were modified in Carson et al.*

***Blanco et al. 2018****: Performed a 3D vs (1D+0D Young) comparison in 32 vessels (20 patients). CCTA and some IVUS data were available, but no FFR measurements. Like Carson et al. the vast majority of the lesions were mild (FFR>0.8).*

***Fossan et al. 2018****: Also performed a 3D vs 1D+0D (Young) comparison in 13 patients. It is novel in that study population featured balanced FFR characteristics (0.46-0.97, 46% positive). Furthermore, uncertainty quantification & sensitivity analysis (UQ&SA) were performed through Monte Carlo sampling that quantified how much the predicted FFR depends on other parameters of the network model. The total coronary resistance index and error in minimal radius were found to be most significant factors.*

***Müller et al. 2019****: This is a follow up study to Fossan et al. It is mainly studying the impact of baseline coronary flow distribution (i.e. outlet BC) on FFR prediction. Excluding the studies assessing Heartflow’s product (which we don’t review here due to undisclosed methodologies), this study was almost unique in its sample size among FFR_CT_ studies, featuring 105 FFR measurements (63 patients). In addition, a 1D+0D (Seely) formulation was compared to 3D CFD and UQ&SA performed. The main conclusions were that BC-related errors are more important for FFR accuracy than ROMs, though there is room for improving ROMs still.*

***Yin et al., 2019****: This is another 3D vs (1D-0D Young) comparison, however featured some unique elements such as using a multifidelity Bayesian optimization to estimate the outlet WK params. This was based on 1 geometry from OSMC with no matching functional measurements.*

***Itu et al., 2016****: This approach broke trend with the other studies in that it trained a neural network to synthetic training data generated by stenoses-containing 1D+0D network models. The trained network was compared with 125 FFR measurements (38 positive) from 87 patients, yielding 83% accuracy.*

*The need for further testing and validation using a patient-specific set up has been added to a new section Limitations and Future Work (L560).*

***vFFR_from angiography***

*The term virtual FFR is more closely associated with angio-based FFR in the literature, possibly due to VIRTU-1 study which kicked off the application. Due to the volume of literature, a complete review of this field is out of scope. We survey the selected highlights below.*

*The vFFR literature occupies a parallel world to FFR_CT_ - They tend to be further along clinical translation (mostly prospective multicentre trials with 100s of samples as standard, and analysed online i.e. while the patient is on the table). All recent papers are clinical trials published in clinical journals. In stark opposition to FFR_CT_ the trajectory of the models has been to progressively go simpler rather than incorporating more modelling components, as computational time is a factor – where 3D CFD is used, it is routinely a rigid walled single vessel, and ways to exploit steady CFD are embraced as an innovation. Some algorithms, including many commercial offerings are simply based on a quadratic equation. For further perspectives, we can recommend editorials by Wong & Yong (2020) and Morris & Gunn (2017), or the recent reviews Gabara et al. (2020) and Morris et al. (2020).*

*Below is a table taken from Gabara et al. (2020) which sums up key vFFR papers – it is noteworthy that none of these papers consider flexible arterial wall, the vast majority use a steady state approximation, and only one method considers the network structure of the vascular network in their model.*

***Morris et al., 2013 (VIRTU-1 trial)****: 35 lesions (19 patients) were modelled and compared to measured FFR. ANSYS CFX was used to simulate unsteady fluid flow in a rigid single vessel or bifurcation geometry with a cohort-averaged windkessel outlet condition. Reported accuracy is high (97%) but the study population featured only about 20% positive FFR and 1 in the grey zone. Offline analysis (~24 hours/case).*

***Papafaklis et al., 2014 (vFAI)****: Caas QCA-3D from Pie Medical Imaging used, steady CFD with fixed inlet pressure and two (rest/hyperemic) flow rates simulated. 139 vessels (60% cases FFR 0.7-0.9), multicentre trial. Difference with wire-FFR was -0.0039±0.085. 15 min processing time/patient. Criticised for not taking in any patient-specific microvascular physiology in its BC estimation but still quite accurate when using vFAI cutoff of 0.82 (88% diagnostic accuracy).*

***Morris et al., 2017 (VIRTU-Fast trial)****: In this follow up work to their 2013 paper, to cut down on simulation time, results of a paired rigid-wall, single vessel, steady simulations were fitted to a simple quadratic equation (which they call pseudotransient model), which was then combined with a distal windkessel model to estimate vFFR. The steady state simulations used a plug velocity profile and uniform zero-pressure outlet (essentially treating the segment as a quadratic resistance). The study featured 73 arteries (29 between 0.7 and 0.9) from 20 patients, computations taking 189s/case. After tuning the distal impedance using invasively measured pressure (i.e. bypassing the practical BC tuning issue), the pseudotransient model was highly accurate wrt measured FFR (mean error ±0.86%). Error in estimating the distal BC was shown to be the dominant factor (59%) compared to error in geometry (33%) for vFFR prediction.*

***Li et al, 2020 (caFFR)****: FLASH-FFR trial – prospective multicentre, FlashAngio software – 3D mesh, TIMI frame to tune BC, proprietary CFD (1 min/vessel), both online and blinded offline caFFR computation. 328 intermediate lesions (119 in greyzone FFR 0.75-0.85), online caFFR 95.7% accuracy (grey zone accuracy 89.9%). <5min per case.*

*Notably, whereas the FFR_CT_ commercial landscape is dominated by a single company, multiple players exist in the vFFR space. Some of the major studies include:*

***Xu et al., 2017 (QFR, Medis)****: Prospective, multicentre trial. 328 vessels (107 in greyzone FFR 0.75-0.85), and currently the most studied angio-derived pressure ratio along with WIFI II trial (Westra, 2018). The analyses were performed online where single vessel lumen contours were automatically segmented. FFR estimation uses a quadratic equation where coefficients are fitted to data and patient-specific mean hyperaemic flow velocity is estimated based on TIMI frame count. FFR difference was -0.01±0.06, and diagnostic accuracy 92.7%, in the intermediate group (FFR 0.75-0.85) 86%. 4.36±2.55 min per patient. The simplifying assumptions underlying the QFR methodology are listed here (from Tu et al, 2016):*

***Fearon et al, 2019 (CathWorks)****: Follow up to Pellicano et al. (2017) - prospective, multicentre international trial. Coronary circulation was treated as an electrical circuit, after angiograms were 3D reconstructed to a tree structure using CathWorks software. Each segment was modelled as a simple resistor, after assessing that Bernoulli forces and other approaches yielded equivalent FFR values. 319 vessels (greyzone proportion not explicitly stated), 92.2% accuracy (87% within greyzone). Average processing time 2.7 minutes/case.*

*Revisions to the paper were made to comment on the different trajectories the FFR_CT_ and vFFR studies have taken in the section Modelling Approaches (L535).*

3) note that FFR is not the only parameter in decision making; several other coefficients have been introduced and studied recently;

*Focusing our discussion on the coronary artery disease, the research community has been very active in the past decade, investigating multiple alternative physiological indices including iFR, IMR, QFR, and a variety of non-hyperaemic pressure ratio indices offered by different vendors (RFR, DFR, dPR, resting pd/pa) to mention a few. However, few are supported at current time by large prospective clinical evidence based on the patient outcome as FFR has been; it is further indicative that the most established of these studies (the NEJM article on iFR) was a non-inferiority trial in comparison with FFR (Goteberg et al,. 2017) illustrating that FFR is still the gold standard against which new indices are measured against.*

*Given the above, we reiterate that the goal of this paper is to design a better-performing reduced order model to help estimate our chosen index, FFR. While some of the alternative indices may perform just as well, or there may be opportunities to use them in conjunction with FFR to enhance diagnostic workflow, these are clearly a matter for clinical trials to sort out. It would be inappropriate within the scope of our simulation-based study to extrapolate our results to the full clinical decision-making algorithm.*

4) check and compare the model's assumptions and realistic physiological conditions (It seems that near transitional turbulent regime hardly occurs in small coronary arteries with constantly collapsible terminals. Probably, the model would be valid for other locations of the stenosis (double-check the patient or general physiological data for that localisations before the next attempt) );

*The diameters of the test geometries in this work range between 4mm to 5cm, which cover the approximate dimensions of a large coronary vessel to aorta. In terms of the coronary vessels, the chosen dimensions are well within the diameters found in the first or second generation vessels measured in CCTA data, such as Left main (3.5±0.8mm), LAD (3.2±0.7mm), LCX (3.0±0.7mm) and RCA (3.4±0.6mm) (Medrano-Gracia et al., 2016). Males tended to have larger calibres than the average figures quoted. Most haemodynamically consequential lesions occur within these large coronary segments, due to their larger distended tissue volume. We agree that transitional flow regime would be of little interest in the small coronary arteries, and we do not consider them in our work. Collapsible terminals are not observed in the epicardial arteries where intramyocardial pressure is significantly lower than in the endocardial regions. The intramural and further branching distal segments subject to such effects are unsuitable as targets for revascularisation, and no stents of suitable dimension would be available.*

5) keep in mind that elastance of the vascular plays a significant role in haemodynamics.

*We thank the reviewer for the scrutiny of rigour in our analysis. We would like to clarify that our reduced order model is designed to characterise the pressure drop across the adjacent proximal and distal regions of vessel segment containing the lesion, and not a whole vessel segment or branches. This is a common basis among all other ROMs we referenced in our work, which allows us to compare the models. And although the modelled CFD domain may extend further distally, this is only to ensure that the numerical BCs posed do not affect the pressure drop characterisation in an uncontrolled manner.*

*In relative terms, the pressure drop across a long healthy portion of the vessel is negligible unless the lesion itself is very mild, in which case the utility of any model for a clinical workflow would be limited. The presence of atherosclerosis is associated with an elevated wall stiffness in the healthy segments as demonstrated by measurements of pulse wave speed (Kim et al., 2019). Add to this the atherosclerotic portion with possible calcifications, it is reasonable to consider that elastance effects will play a limited role in the localised trans-stenotic pressure drop. Conversely, simulation studies which have employed elastic walled flow models consistently report a limited role of the vascular elastance on pressure drop (2D, Ventre et al., 2019; 3D FSI, Mirramezani et al, 2019). If you are aware of evidence that suggests otherwise, we would be grateful to be introduced to it.*

An excerpt from Mirramezani et al (2019)

Figure 3 from Ventre et al., 2019. Note the two 2D models, the Multi-ring model (featuring elastic vessel wall) and RNSP (rigid wall) are almost identical.

*This is, of course, not to say that elastance effects do not matter at all – focusing on coronary flow, they must be taken into account when one considers e.g. wave propagations in the wider vascular system. For network modelling, commonly the lesion model (whether it be a ROM, or else) is inserted within an elastic 1D or 3D coronary network that addresses the elastance, employing a multiscale approach to optimise on computational cost (please also refer to our survey of FFR_CT_ study in #2).*

***References***

*Arthurs, C. J., Khlebnikov, R., Melville, A., Marčan, M., Gomez, A., Dillon-Murphy, D., ... & Figueroa, C. A. (2021). CRIMSON: An open-source software framework for cardiovascular integrated modelling and simulation. PLOS Computational Biology, 17(5), e1008881.*

*Blanco, P. J., Bulant, C. A., Müller, L. O., Talou, G. M., Bezerra, C. G., Lemos, P. A., & Feijóo, R. A. (2018). Comparison of 1D and 3D models for the estimation of fractional flow reserve. Scientific reports, 8(1), 1-12.*

*Boileau, E., Pant, S., Roobottom, C., Sazonov, I., Deng, J., Xie, X., & Nithiarasu, P. (2018). Estimating the accuracy of a reduced‐order model for the calculation of fractional flow reserve (FFR). International journal for numerical methods in biomedical engineering, 34(1), e2908.*

*Buoso, S., Manzoni, A., Alkadhi, H., Plass, A., Quarteroni, A., & Kurtcuoglu, V. (2019). Reduced-order modeling of blood flow for noninvasive functional evaluation of coronary artery disease. Biomechanics and modeling in mechanobiology, 18(6), 1867-1881.*

*Carson, J. M., Pant, S., Roobottom, C., Alcock, R., Javier Blanco, P., Alberto Bulant, C., ... & Nithiarasu, P. (2019). Non‐invasive coronary CT angiography‐derived fractional flow reserve: A benchmark study comparing the diagnostic performance of four different computational methodologies. International journal for numerical methods in biomedical engineering, 35(10), e3235.*

*Cantwell C. D., D. Moxey, A. Comerford, A. Bolis, G. Rocco, G. Mengaldo, D. De Grazia, S. Yakovlev, J.-E. Lombard, D. Ekelschot, et al., Nektar++: An open-source spectral/hp element framework, Computer physics communications 192 (2015) 205–219.*

*Epstein, S., Willemet, M., Chowienczyk, P. J., & Alastruey, J. (2015). Reducing the number of parameters in 1D arterial blood flow modeling: less is more for patient-specific simulations. American Journal of Physiology-Heart and Circulatory Physiology, 309(1), H222-H234.*

*Fearon, W. F., Achenbach, S., Engstrom, T., Assali, A., Shlofmitz, R., Jeremias, A., ... & FAST-FFR Study Investigators. (2019). Accuracy of fractional flow reserve derived from coronary angiography. Circulation, 139(4), 477-484.*

*Fossan, F. E., Sturdy, J., Müller, L. O., Strand, A., Bråten, A. T., Jørgensen, A., ... & Hellevik, L. R. (2018). Uncertainty quantification and sensitivity analysis for computational FFR estimation in stable coronary artery disease. Cardiovascular engineering and technology, 9(4), 597-622.*

*Gabara, L., Hinton, J., Gunn, J., Morris, P. D., & Curzen, N. (2020). Coronary Physiology Derived from Invasive Angiography: Will it be a Game Changer?. Interventional Cardiology Review, 15.*

*Garcia, D., Pibarot, P., and Duranda, L. G., 2005, “Analytical Modeling of the Instantaneous Pressure Gradient Across the Aortic Valve,” J. Biomech., 38(6), pp. 1303–1311.*

*Götberg, M., Christiansen, E. H., Gudmundsdottir, I. J., Sandhall, L., Danielewicz, M., Jakobsen, L., ... & Fröbert, O. (2017). Instantaneous wave-free ratio versus fractional flow reserve to guide PCI. New England Journal of Medicine, 376(19), 1813-1823.*

*Huo, Y. L., Svendsen, M. J., Choy, S., Zhang, Z. D., and Kassab, G. S., 2012, “A Validated Predictive Model of Coronary Fractional Flow Reserve,” J. R. Soc., Interface., 9(71), pp. 1325–1338.*

*Itu, L., Rapaka, S., Passerini, T., Georgescu, B., Schwemmer, C., Schoebinger, M., ... & Comaniciu, D. (2016). A machine-learning approach for computation of fractional flow reserve from coronary computed tomography. Journal of applied physiology, 121(1), 42-52.*

*Kim, H. J., Vignon-Clementel, I. E., Coogan, J. S., Figueroa, C. A., Jansen, K. E., & Taylor, C. A. (2010). Patient-specific modeling of blood flow and pressure in human coronary arteries. Annals of biomedical engineering, 38(10), 3195-3209.*

*Kim, H. L., & Kim, S. H. (2019). Pulse wave velocity in atherosclerosis. Frontiers in cardiovascular medicine, 6, 41.*

*Kim, J., Jin, D., Choi, H., Kweon, J., Yang, D. H., & Kim, Y. H. (2020). A zero-dimensional predictive model for the pressure drop in the stenotic coronary artery based on its geometric characteristics. Journal of Biomechanics, 113, 110076.*

*Lee J., A. Cookson, I. Roy, E. Kerfoot, L. Asner, G. Vigueras, T. Sochi, S. Deparis, C. Michler, N. P. Smith, et al., Multiphysics computational modeling in cheart, SIAM Journal on Scientific Computing 38 (2016) C150–C178.*

*Li, J., Gong, Y., Wang, W., Yang, Q., Liu, B., Lu, Y., ... & Huo, Y. (2020). Accuracy of computational pressure-fluid dynamics applied to coronary angiography to derive fractional flow reserve: FLASH FFR. Cardiovascular research, 116(7), 1349-1356.*

*Medrano-Gracia, P., Ormiston, J., Webster, M., Beier, S., Young, A., Ellis, C., ... & Cowan, B. (2016). A computational atlas of normal coronary artery anatomy. EuroIntervention: journal of EuroPCR in collaboration with the Working Group on Interventional Cardiology of the European Society of Cardiology, 12(7), 845-854.*

*Mirramezani, M., Diamond, S. L., Litt, H. I., & Shadden, S. C. (2019). Reduced order models for transstenotic pressure drop in the coronary arteries. Journal of biomechanical engineering, 141(3).*

*Morris, P. D., Ryan, D., Morton, A. C., Lycett, R., Lawford, P. V., Hose, D. R., & Gunn, J. P. (2013). Virtual fractional flow reserve from coronary angiography: modeling the significance of coronary lesions: results from the VIRTU-1 (VIRTUal Fractional Flow Reserve From Coronary Angiography) study. JACC: Cardiovascular Interventions, 6(2), 149-157.*

*Morris, P. D., & Gunn, J. P. (2017). Computing Fractional Flow Reserve From Invasive Coronary Angiography: Getting Closer.*

*Morris, P. D., Silva Soto, D. A., Feher, J. F., Rafiroiu, D., Lungu, A., Varma, S., ... & Gunn, J. P. (2017). Fast virtual fractional flow reserve based upon steady-state computational fluid dynamics analysis: results from the VIRTU-Fast study. JACC: Basic to Translational Science, 2(4), 434-446.*

*Morris, P. D., Curzen, N., & Gunn, J. P. (2020). Angiography‐Derived Fractional Flow Reserve: More or Less Physiology?. Journal of the American Heart Association, 9(6), e015586.*

*Müller, L. O., Fossan, F. E., Bråten, A. T., Jørgensen, A., Wiseth, R., & Hellevik, L. R. (2019). Impact of baseline coronary flow and its distribution on fractional flow reserve prediction. International journal for numerical methods in biomedical engineering, e3246.*

*Papafaklis, M. I., Muramatsu, T., Ishibashi, Y., Lakkas, L. S., Nakatani, S., Bourantas, C. V., ... & Serruys, P. W. (2014). Fast virtual functional assessment of intermediate coronary lesions using routine angiographic data and blood flow simulation in humans: comparison with pressure wire-fractional flow reserve. EuroIntervention, 10(5), 574-583.*

*Schrauwen, J. T., Koeze, D. J., Wentzel, J. J., van de Vosse, F. N., van der Steen, A. F., & Gijsen, F. J. (2015). Fast and accurate pressure-drop prediction in straightened atherosclerotic coronary arteries. Annals of biomedical engineering, 43(1), 59-67.*

*Seeley, B. D., and Young, D. F., 1976, “Effect of Geometry on Pressure Losses Across Models of Arterial Stenosis,” J. Biomech., 9(7), pp. 439–448.*

*Yin, M., Yazdani, A., & Karniadakis, G. E. (2019). One-dimensional modeling of fractional flow reserve in coronary artery disease: Uncertainty quantification and Bayesian optimization. Computer Methods in Applied Mechanics and Engineering, 353, 66-85.*

*Ventre, J., Raimondi, F., Boddaert, N., Fullana, J. M., & Lagrée, P. Y. (2019, August). Reduced-order models for blood pressure drop across arterial stenoses. In International Symposium on Computer Methods in Biomechanics and Biomedical Engineering (pp. 1-15). Springer, Cham.*

*Weller H. G., G. Tabor, H. Jasak, C. Fureby, A tensorial approach to computational continuum mechanics using object-oriented techniques, Comput. Phys. 12 (1998) 620–631.*

*Wong, C. C., & Yong, A. S. (2020). Flash-forward: the emergence of angiography-derived fractional flow reserve in the catheter laboratory.*

*Xu, B., Tu, S., Qiao, S., Qu, X., Chen, Y., Yang, J., ... & Hu, S. (2017). Diagnostic accuracy of angiography-based quantitative flow ratio measurements for online assessment of coronary stenosis. Journal of the American College of Cardiology, 70(25), 3077-3087.*

*Young, D. F., Cholvin, N. R., & Roth, A. C. (1975). Pressure drop across artificially induced stenoses in the femoral arteries of dogs. Circulation research, 36(6), 735-743.*
