# Supplementary material for: An improved reduced-order model for pressure drop across arterial stenoses
Source: PLoS One. 2021 Oct 1;16(10):e0258047. doi: 10.1371/journal.pone.0258047 (PMC8486142; doi:10.1371/journal.pone.0258047)
Supplement: Attachment — Submitted filename: Response_to_Reviewers.doc [file pone.0258047.s003.doc]

**Reviewer(s)' Comments to Author:**

**Reviewer: 2**

Thank you for the detailed answer. The required reviews are not for me, but for the authors and for the readers. Unfortunately, a lot of the discussion in the reply was not revealed in the text. Yet, I suggest the changes are sufficient for accepting the paper for publication.

***Author’s response:***

Thank you for the valuable comments. We have added a new section “Physiological and Clinical Relevance” towards the end of our Discussion in lines 561-586. In it, we bring together the issues explored in #3, #4 and #5 of our original response to the comments of the reviewer that were not explicitly included in the text before. As #1 and #2 were covered in our previous iteration, we believe that all relevant issues raised are now fully addressed in the text.
